# Differential regulation of lymphatic junctional morphology and the potential effects on cardiovascular diseases

**DOI:** 10.3389/fphys.2023.1198052

**Published:** 2023-04-28

**Authors:** Amanda M. Peluzzo, Meriem Bkhache, Long Nguyen Hoang Do, Michael V. Autieri, Xiaolei Liu

**Affiliations:** Department of Cardiovascular Sciences, Lemole Center for Integrated Lymphatics Research, Temple University Lewis Katz School of Medicine, Philadelphia, PA, United States

**Keywords:** lymphatics, permeability, lymph flux, junctions, VE-cadherin, angiopoietin-2, atherosclerosis, cardiovascular disease

## Abstract

The lymphatic vasculature provides an essential route to drain fluid, macromolecules, and immune cells from the interstitium as lymph, returning it to the bloodstream where the thoracic duct meets the subclavian vein. To ensure functional lymphatic drainage, the lymphatic system contains a complex network of vessels which has differential regulation of unique cell-cell junctions. The lymphatic endothelial cells lining initial lymphatic vessels form permeable “button-like” junctions which allow substances to enter the vessel. Collecting lymphatic vessels form less permeable “zipper-like” junctions which retain lymph within the vessel and prevent leakage. Therefore, sections of the lymphatic bed are differentially permeable, regulated in part by its junctional morphology. In this review, we will discuss our current understanding of regulating lymphatic junctional morphology, highlighting how it relates to lymphatic permeability during development and disease. We will also discuss the effect of alterations in lymphatic permeability on efficient lymphatic flux in health and how it may affect cardiovascular diseases, with a focus on atherosclerosis.

## 1 Introduction

The lymphatic vasculature runs in parallel to the blood vasculature with distinct structural and functional roles that differ from blood vessels. The blood vasculature is a bidirectional circulatory system where blood and nutrients are transported both into and away from organs. Conversely, the lymphatic vasculature is a unidirectional circulatory system that drains lymph (containing fluid, macromolecules, antigens, and immune cells) away from organ tissue to return it to the blood circulation (Alitalo, 2011; Oliver et al., 2020; Liu and Yu, 2022). Although one continuous vessel, the lymphatic vasculature consists of two structurally and functionally unique vessel morphologies: lymphatic capillaries (initial lymphatics) and collecting lymphatic vessels (collecting lymphatics). Both initial lymphatics and collecting lymphatics are lined with lymphatic endothelial cells (LECs), which are connected by cell-cell junctions. However, these junctions are structurally different to meet the unique functions of these two vessel subtypes.

The lymphatic system begins as blind-ended, finger-like initial lymphatics, which are present in the parenchyma of many organ tissues (Alitalo, 2011; Oliver et al., 2020). Since initial lymphatic vessels are responsible for uptake of fluid, macromolecules, and cells into the lymphatic system, LECs lining the initial lymphatics are loosely connected to form “button-like” junctions which, analogous to buttons on clothing, allow for movement of substances through the flaps generated by the overlapping cell borders in the gaps between them (Baluk et al., 2007; Baluk and McDonald, 2022; Scallan and Jannaway, 2022). These flaps function as a valve, allowing various substances to enter the lymphatic vessel while also preventing the leakage of those substances (Tammela and Alitalo, 2010; Oliver et al., 2020; Scallan and Jannaway, 2022). Studies have also revealed that LECs are connected to the extracellular matrix by anchoring filaments, so elevated interstitial fluid could expand the tissue and therefore aid in fluid and cell entry by opening the flaps (Leak and Burke, 1966; Leak, 1970). Initial lymphatics coalesce to form collecting lymphatics which then carry lymph through draining lymph nodes, making their way to the thoracic duct and finally drain into the subclavian vein (Alitalo et al., 2005; Alitalo, 2011; Oliver et al., 2020).

Collecting lymphatics are different from the hyper-permeable initial lymphatics. They share similar features to blood vessels, with a thin layer of specialized smooth muscle cells and bi-leaflet valves that together contribute to lymphatic fluid flux (Alitalo et al., 2005; Oliver et al., 2020). Importantly, LECs lining collecting lymphatics have their junctions organized as continuous “zippers” which, similar to zippers on clothing, form a tight barrier to promote functional lymph transport (Baluk et al., 2007; Baluk and McDonald, 2022; Scallan and Jannaway, 2022). Although the collecting vessels form a tighter barrier than initial vessels, they are not completely impermeable (Breslin et al., 2018; Scallan and Jannaway, 2022). In fact, collecting lymphatics have passive permeability comparable to that of blood capillaries (Scallan and Jannaway, 2022).

Therefore, the lymphatic system must initially be permeable enough to receive substances from organ tissues, but later become a barrier to prevent leakage so substances that form lymph are maintained within the vessel (Oliver et al., 2020; Scallan and Jannaway, 2022). A growing body of evidence has shown that lymphatic function is tightly controlled by dynamic and differential regulation of junctional morphology during development and diseases (Breslin et al., 2018; Baluk and McDonald, 2022; Scallan and Jannaway, 2022). The functional relevance of this regulation during the pathogenesis of cardiovascular disease has only just recently been appreciated. This review will mainly focus on current studies regarding lymphatic vessel function and lymph flux as it relates to junctional morphology and lymphatic permeability in both physiological and diseased states.

## 2 Junctional morphology and lymph flux

The differential regulation of lymphatic junctional morphology and how it relates to lymph flux is a core, yet understudied topic in lymphatic research. First, it is important to understand a few key terms in the context of the lymphatic system: permeability and lymph flux. Scallan and Jannaway eloquently described the definition of permeability as a measurement of the rate at which particles, both solute and solvent, can pass through a semi-permeable membrane (Scallan and Jannaway, 2022). From the perspective of a vascular biologist, permeability is often viewed from a negative perspective. For example, blood vascular permeability can contribute to cancer metastasis (Tomita et al., 2021), diabetic retinopathy (Rudraraju et al., 2020), and increase the likelihood of mortality in sepsis patients (Yang et al., 2022). On the other hand, lymphatic vascular permeability is vital for lymphatic vessel function to maintain tissue homeostasis through the removal of fluid, macromolecules, antigens, and immune cells that comprise lymph fluid (Alitalo et al., 2005; Liu and Yu, 2022; Scallan and Jannaway, 2022). However, the lymphatic vasculature is not permeable in its entirety. As noted earlier, the main permeable segment of a lymphatic vessel is the initial lymphatic vessel while collecting lymphatic vessels are less permeable to maintain lymph within the system and propel it. This maintenance and subsequent movement of lymph fluid within a lymphatic vessel is termed lymph flux (Hansen et al., 2015).

Past studies have focused on analyzing the role of collecting vessel contraction and valvular function, which led to interesting discoveries on lymphatic flux in many diseases (Scallan et al., 2016; Liao et al., 2019; Milasan et al., 2019; Lee et al., 2022), but fewer studies focus on lymphatic junctional morphology as it relates to lymph flux in health and disease. Such studies focusing on lymphatic junctional regulation are vital because junctions help maintain the function and integrity of lymphatic vessels. Without buttons, initial lymphatics would likely be less permeable, preventing lymph from entering the vessel. Conversely, without zippers, collecting lymphatics would be too permeable and less likely to keep lymph within the vessel. For example, one study showed that vascular endothelial growth factor A (VEGF-A) dependent zippering of lacteals (initial lymphatics within the small intestine) prevents lymphatic uptake of chylomicrons (Zhang et al., 2018). On the other hand, vessel integrity is disrupted in diabetic (*db/db*) mice which led to increased permeability of collecting lymphatic vessels and dye leakage from the vessel (Scallan et al., 2015). It is also important to note that there are differences in lymphatic function between lymphatic beds of different organs, particularly junctional morphology as it relates to permeability and lymph flux (O'Morchoe and O'Morchoe, 1987; den Braanker et al., 2021). Adding complexity to the concept of permeability, we cannot ignore the transcellular pathway where substances enter lymphatics through vesicular transport, opposed to paracellular transport (Liu and Yu, 2022). One study determined that the paracellular pathway between button junctions is non-selective while the transcellular pathway is more selective (Scallan and Jannaway, 2022). Nonetheless, it is essential to study the spatial regulation of lymphatic permeability as it relates to effective lymph flux to truly understand its impact in health and disease.

## 3 Junctional regulation during lymphatic development

There are many junctional proteins that contribute to the formation of both button and zipper morphology. These proteins include that of adherens junctions (vascular endothelial cadherin (VE-cadherin), β-catenin, and p120-catenin) and that of tight junctions (occludin, claudin-5, and ZO-1) (Baluk et al., 2007; Yao et al., 2012), which were all found to have a similar distribution throughout lymphatic development (Yao et al., 2012). Despite the fact tight junctional proteins are present at lymphatic junctions and are critical for lymphatic integrity, less is known about the effects of tight junctional proteins on the formation of buttons and/or zippers (Zhang et al., 2020). Conversely, VE-cadherin is well studied and has been shown to play a critical role in junctional formation and maintenance in both blood vessels and lymphatic vessels (Baluk et al., 2007; Dejana et al., 2009; Yao et al., 2012; Zhang et al., 2020). Moreover, lymphatic-specific deletion of VE-cadherin leads to severe edema and lethality by E14.5 (Hägerling et al., 2018). When VE-cadherin is deleted postnatally, dermal lymphatics have a compensatory upregulation of tight junction proteins and are largely unaffected, while intestinal and mesenteric lymphatics are defective (Hägerling et al., 2018). For a more thorough review of lymphatic junctional proteins, see Zhang et al. (Zhang et al., 2020).

From the first bud that generates the lymphatic sprout from the cardinal vein at E10.5-E12.5, lymphatic endothelial cell junctions present with zipper morphology in the initial lymphatic vessels (Yang et al., 2012). This zipper morphology starts to convert to button morphology in the initial lymphatics at E17.5 of the trachea and diaphragm (Yao et al., 2012; Zheng et al., 2014). The initial lymphatics obtain nearly 100% button junctional morphology by P70 (Yao et al., 2012). Ubiquitous button formation in initial lymphatic vessels was expedited to occur by P4 in mice treated with dexamethasone due to direct actions on LEC glucocorticoid receptors (Yao et al., 2012). However, treatment with antibodies against Angiopoietin-2 (Angpt2) was able to prevent dexamethasone-associated button formation, identifying Angpt2 as a crucial molecule in the remodeling of zippers to buttons in initial lymphatics during development (Zheng et al., 2014). In addition, Angpt2^−/−^ mice had junctions resembling zippers in the initial lymphatics of the diaphragm while also having disrupted junctions resembling buttons in collecting lymphatics of the ear and mesentery, resulting in impaired lymph uptake (Zheng et al., 2014).

Since adrenomedullin signaling is recognized as an important factor in developmental lymphangiogenesis and zipper formation, researchers have sought to identify its downstream effectors (Xu et al., 2018). Rap1 (Ras-related protein) is a GTPase involved in strengthening the endothelial barrier (Fukuhara et al., 2005). Lymphatic-specific deletion of *Rap1a/b* isoforms led to edema and abnormal junctional morphology in the jugular lymph sac during development (Xu et al., 2018). Inducible deletion of *Rap1a/b* at 3 months of age led to discontinuous button junctions and leakage of Evans blue dye from collecting lymphatics despite treatment with adrenomedullin (Xu et al., 2018). In addition, lymphatic-specific deletion of *Rasip1*, an endothelial-specific regulator of GTPases, leads to edema, dilated lymphatics, and altered cell junctions (Liu et al., 2018). Upon analysis of junctional morphology, *Rasip1* conditional knockout embryos were found to have discontinuous adherens junctions (VE-cadherin) and tight junctions (ZO-1) in the dermis, lung, and mesenteric initial lymphatics compared to developing initial lymphatics in control mice which had characteristic zipper formation throughout development (Liu et al., 2018). Subsequent *in vitro* experiments indicate that Rasip1 regulates Cdc42 GTPase activity which promotes endothelial cell focal adhesions to extracellular matrix (Liu et al., 2018). Recent work also identified several other major signaling pathways that control tight junction localization and lymphatic vessel integrity, including EphrinB2/EphB4 and S1PR1 (Frye et al., 2020; Geng et al., 2020). This emphasizes the intricate regulation of junctional morphology throughout development, starting as zippered junctions during developmental lymphangiogenesis and eventually converting to functional button junctions in initial lymphatics.

## 4 Junctional regulation during inflammation

Inflammation-associated lymphangiogenesis may be similar to developmental lymphangiogenesis in that the sprouting tips of new vessels are zippered, but the molecular regulation of this process is much less understood (Liao and von der Weid, 2014). It has been well documented that inflammation triggers blood vessel leakage by inducing microvascular changes, including formation of gaps between blood endothelial cells (Baluk and McDonald, 1994; McDonald, 1994; McDonald, 2001). However, lymphatic vessels seem to experience the opposite effect. While initial lymphatic vessels are hyper-permeable with discontinuous button junctions in the healthy state, current studies have shown that these junctions undergo a button-to-zipper transformation during inflammatory diseases. The following studies utilize infection-based techniques to induce inflammation through bacteria, bacterial byproducts, or viruses. However, various pathological processes are also inflammatory, including but not limited to cardiovascular diseases. Further research is needed to determine if the following findings are also applicable under various inflammatory diseases. Under exposure to the bacteria *Mycoplasma pulmonis*, the airways become inflamed, and inflammation-associated lymphangiogenesis ensues (Baluk et al., 2007; Yao et al., 2012). The newly formed lymphatic sprouts are connected by zipper junctions instead of button junctions (Baluk et al., 2007; Yao et al., 2012). These continuous junctions appear to be less permeable, resulting in impaired fluid entry and drainage (Yao et al., 2012). Hence, alterations to the typical initial lymphatic junctional morphology during inflammation could alter lymphatic drainage function, contributing to mucosal edema and airflow obstruction. Interestingly, treatment of the airway infection with dexamethasone, an anti-inflammatory corticosteroid, promotes lymphatic zipper-to-button conversion through direct actions on LECs (Yao et al., 2012). Therefore, it is possible that changes in lymphatic endothelial junctional dynamics during inflammation could be reverted to homeostatic conditions through drug intervention, restoring proper lymphatic function and improving lymph clearance. Although it is uncertain if inflammation-associated lymphangiogenesis contributes to disease progression or is simply a failed attempt at disease resolution, the above study suggests it is possibly the latter. Lymphangiogenesis may be occurring in attempt to remove pro-inflammatory substances from the organ tissue, but the inflammatory environment may be preventing lymphatic function by promoting initial lymphatic zippering. Future studies should evaluate the role of other anti-inflammatory agents in promoting lymphatic vessel function through maintaining the structural integrity of both initial and collecting lymphatics, allowing proper lymph flux to prevent disease progression.

In addition to fluid transport, inflammation-driven lymphatic button-to-zipper transformation may also alter transport of immune cells and macromolecules. A recent study using a mouse model for chronic inflammation of the lung pleura has also confirmed junctional zippering during inflammation. In this study, intrathoracic injections of *Escherichia coli* lipopolysaccharide (LPS) led to increased lymphangiogenesis with zippered initial lymphatic vessels in the pleural side of the diaphragm (Ngamsnae et al., 2023). Treatment with axitinib, a vascular endothelial growth factor receptor (VEGFR) inhibitor, after LPS challenge rescues LPS-induced junctional changes and preserves lymphatic drainage function (Ngamsnae et al., 2023). Axitinib was also shown to reduce the amount of infiltrating white blood cells, which may also be a result of increased lymphatic drainage leading to egression of inflammatory cells. Considering the fact that axitinib is a pan-inhibitor for all VEGFRs, including VEGFR-1, -2, and -3, this study also determined alterations in angiogenesis and blood vessel permeability upon axitinib treatment of LPS-challenged mice (Siedlecki et al., 2017; Ngamsnae et al., 2023). Future studies using molecules to specifically target VEGFR3, which is more specific to LECs, may determine the contribution of lymphatic function in LPS-induced airway inflammation. Another recent study showed that vaccinia virus infection through scarification induces dermal lymphatic button-to-zipper transformation which restricts viral dissemination and dendritic cell egression through lymphatic vessels (Churchill et al., 2022). Interestingly, VEGF-A levels were elevated in the skin upon viral infection, leading to increased VEGF-A/VEGFR2 signaling which has previously been shown to contribute to initial lymphatic zippering (Zhang et al., 2018; Churchill et al., 2022).

Despite various studies showing VEGF-A as an important molecular cue for lymphatic zippering (Zhang et al., 2018; Zhang et al., 2020; Churchill et al., 2022), the effects of VEGF-A in lymphatic junctional remodeling appear to be organ-specific, as VEGF-A signaling was not critical in remodeling of lymphatics after bacterial induced airway inflammation (Baluk et al., 2009), even though there is significant initial lymphatic vessel zippering (Yao et al., 2012). Nevertheless, future studies are needed to further confirm whether VEGF-A is elevated in organ-specific inflammation to determine such a role. Furthermore, whether additional molecules are responsible for immune cell egression through lymphatics is still an open question. Although some studies note dampened white blood cell egression associated with lymphatic zippering during inflammation (Churchill et al., 2022), one study identified rapid dendritic cell migration during inflammation, but they did not examine junctional morphology during the inflammatory process (Arasa et al., 2021).

**FIGURE 1:**
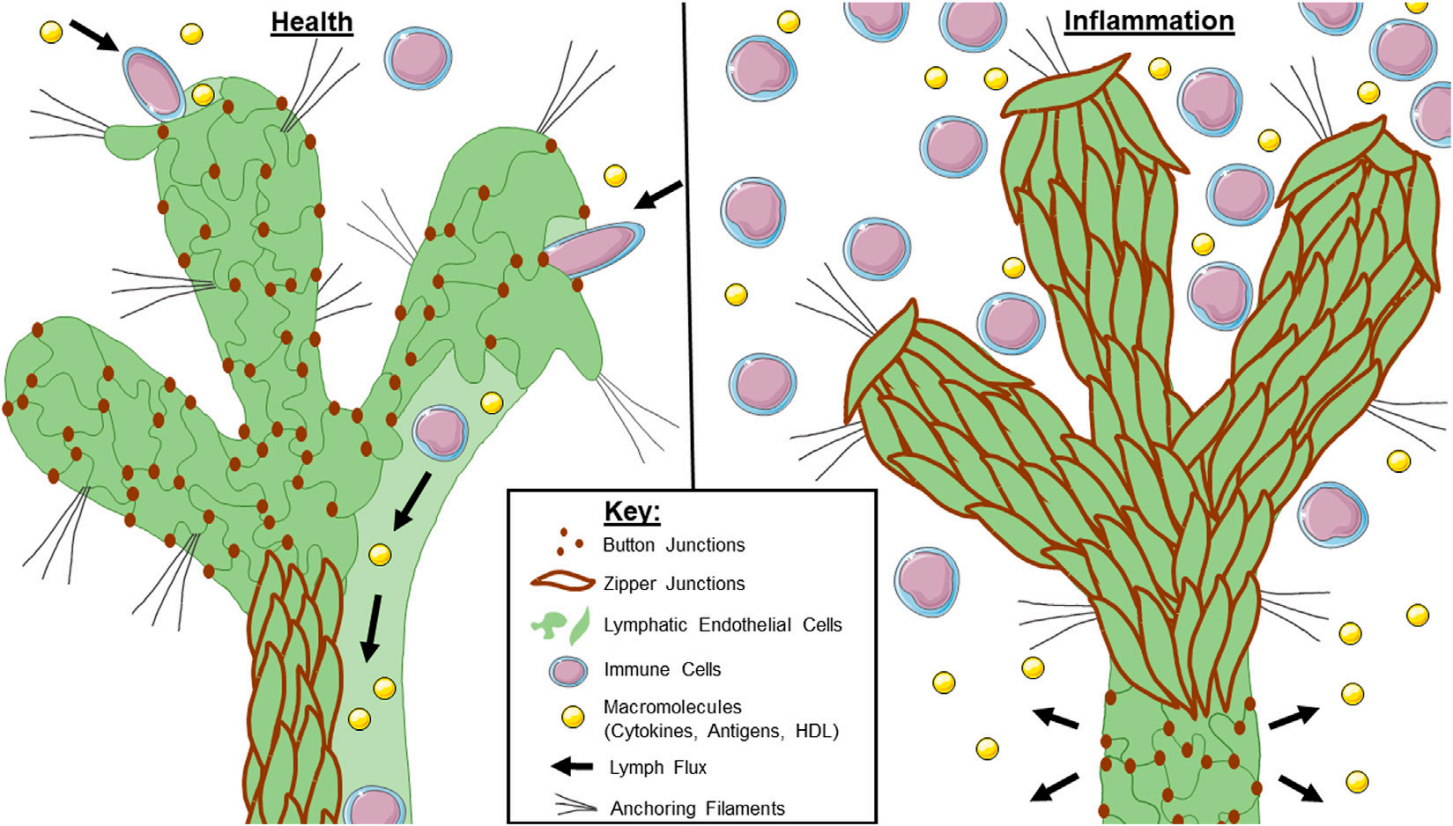
Lymphatic junctional morphology during health and inflammation. In a healthy lymphatic vessel (left), the initial lymphatics are lined with button junctions. As interstitial pressure increases, the anchoring filaments expand with the extracellular matrix which facilitates lymph entry through the gaps between buttons. Lymph is maintained within the lymphatic system by virtue of the zipper junctions in the collecting lymphatics. During inflammation (right), initial lymphatics undergo a button-to-zipper transformation which may prevent lymph uptake. Less is known about the junctional morphology of collecting lymphatics during inflammation, but some studies suggest zipper-to-button transformation which would cause leakage of any lymph that makes it into the system. Overall, this contributes to poor lymphatic flux during inflammation.

Much less is known about collecting lymphatic vessel junctions during inflammation and conflicting results exist in the literature. Although bacterial induced airway inflammation altered initial lymphatic junctional morphology, collecting lymphatic junctions remained unaffected and continually zippered (Yao et al., 2012). In another study, the bacteria *Yersinia pseudotuberculosis* induced inflammation of the mesenteric collecting vessels and exhibited increased permeability, possibly due to a zipper-to-button transformation (Fonseca et al., 2015). Consistent with this study, *in vitro* experiments on LECs isolated from mesenteric collecting vessels exhibited increased permeability when treated with pro-inflammatory cytokines such as TNF-α, IL-6, IFN-λ, and LPS (Cromer et al., 2014). As stated earlier, if initial lymphatics undergo button-to-zipper transformation, less lymph will enter the lymphatic system. If collecting lymphatics undergo the converse zipper-to-button transformation, any lymph within the system could extravasate, adding an additional parameter to decrease lymphatic flux efficiency. Further studies, both *in vitro* and *in vivo*, are vital to determine the conditions in which collecting vessel permeability is affected *versus* initial vessels. Since commercially available LECs used for culture are a mixture of endothelial cells from both initial and collecting lymphatic vessels, it would be difficult to parse out differential regulation of junctions *in vitro* (Scallan and Jannaway, 2022). Therefore, *in vivo* experiments may be more valuable. The molecular mechanisms driving lymphatic junctional regulation of both initial and collecting lymphatic vessels should be evaluated in the context of each disease of interest, looking at tissue-specific lymphatic function. See Figure 1 for a summary of lymphatic junctional changes in health and disease.

## 5 Molecular regulation of lymphatic junctions

Generally, blood endothelial cells express complexes of VE-cadherin and VEGFR2, and VEGF-A activation leads to phosphorylation of tyrosine 949, c-Src activation, and internalization of the receptor, increasing blood vascular permeability (Li et al., 2016; Heinolainen et al., 2017). Although the universal role of VEGF-A in lymphatic junctional patterning is ambiguous in terms of airway lymphatics, Zhang et al. determined its role in lacteals. They performed genetic deletion of VEGF-A scavenger receptors Neuropilin1 (Nrp1) and Vascular endothelial growth factor receptor 1 (Flt1) (Zhang et al., 2018). In the absence of these scavenger receptors, VEGF-A bioavailability increases in the interstitium to drive VEGFR2 signaling, preventing VE-cadherin cytoskeletal anchoring, thereby inducing the zippering of tight junctions of the initial lymphatic vessels known as lacteals (Zhang et al., 2018). This prevented uptake of chylomicrons, the major macromolecule transported through the intestinal lymphatic system (Zhang et al., 2018; Zhang et al., 2020). The authors also determined that inhibition of Rho guanosine triphosphatase (GTPase) signaling similarly decreased VE-cadherin anchoring to the cytoskeleton while inducing zipper formaiton, concluding that cytoskeletal anchoring may induce a force to maintain button formation (Zhang et al., 2018). Although lymphatic zippering in Nrp1/Flt1 mutant mice occurred exclusively in lymphatic lacteals of the intestine, the authors found that exogenous administration of VEGF-A, both intravascularly and intradermally, led to dermal lymphatic zippering (Zhang et al., 2018). Therefore, the lymphatic lacteals may be more sensitive to nuanced changes in VEGF-A levels induced by deletion of Nrp1/Flt1, whereas lymphatic vessels in other tissues require more striking changes in VEGF-A to alter junctional morphology.

There also seems to be a role for VEGF-C/VEGFR3 in the regulation of lymphatic junctions during adulthood (Tacconi et al., 2015; Heinolainen et al., 2017). Interestingly, the following permeability data seems to contradict both developmental and VEGF-C induced adult lymphangiogenesis which has been previously associated with zippering in the sprouts of newly formed lymphatic vessels (Yang et al., 2012; Yao et al., 2012; Zheng et al., 2014). One study, focusing on blood vascular permeability, genetically deleted VEGFR3 in blood endothelial cells which led to upregulated VEGFR2 activity and blood vascular permeability (Heinolainen et al., 2017). This study concluded that VEGFR3 regulates VEGFR2 activation and therefore plays a role in permeability, albeit in blood vessels (Heinolainen et al., 2017). Another study determined a role for the VEGF-C/VEGFR3 pathway in regulating lymphatic junctional proteins to promote colorectal cancer metastasis (Tacconi et al., 2015). They determined that VEGF-C treatment, in both healthy and tumor-bearing mice, led to a discontinuous VE-cadherin distribution resembling buttons while VEGFR3-blocking antibodies partially restored canonical button/zipper distributions in the colonic lymphatic vessels (Tacconi et al., 2015). *In vitro*, they determined that VEGF-C/VEGFR3 signaling led to disassembly of the VE-cadherin/β-catenin complex, VE-cadherin internalization, and increased permeability leading to enhanced transendothelial migration of cancer cells (Tacconi et al., 2015). Another study in cardiac lymphatics demonstrated that loss of VE-cadherin results in VEGFR3 internalization and diminished signaling (Harris et al., 2022). In addition, Hong et al. determined VEGF-C/VEGFR3 signaling may play an important role in maintaining lacteal button morphology and dietary fat uptake (Hong et al., 2020). They showed that intestinal stromal cells increase VEGF-C secretion through YAP/TAZ activation, leading to maintenance of lacteal function (Hong et al., 2020). Subsequent depletion of YAP/TAZ signaling led to less VEGF-C secretion, increased lacteal zippering, and reduced lipid uptake (Hong et al., 2020).

And yet, an opposing study found that VEGF-C induced continuous zipper junctions in human dermal LECs *in vitro* by acting on VEGFR2, like VEGF-A (Cifarelli et al., 2021). This zippering was attenuated when CD36, a transmembrane scavenger receptor, was knocked down with siRNA which reportedly also attenuated VEGF-C signaling through VEGFR2 (Cifarelli et al., 2021). This contradicts the previous studies reporting VEGF-C as a molecule regulating initial lymphatic button formation. Keep in mind, *in vitro* experiments would consist of endothelial cells from both initial and collecting lymphatic vessel segments, making it difficult to determine relevance to an *in vivo* system. Remarkably, lymphatic-specific deletion of CD36 in a mouse model led to discontinuous VE-cadherin junctions in collecting mesenteric lymphatic vessels and impaired lymphatic flux, indicated by leakage of BODIPY into the surrounding mesenteric tissue (Cifarelli et al., 2021). Therefore, VEGF-C/VEGFR2 signaling, regulated through CD36, may play a stronger role in collecting lymphatic zipper maintenance while VEGF-C/VEGFR3 signaling may be maintaining initial lymphatic buttons. That said, VEGF-C has a higher affinity for VEGFR3, but VEGFR3 signaling was not evaluated in this study (Shibuya, 2011; Cifarelli et al., 2021). Therefore, junctional regulation through VEGF-C signaling should be evaluated *in vivo* through VEGF-C156S which displays further reduced affinity for VEGFR2, making studies more applicable to the VEGFR3 pathway (Dieterich et al., 2017). In addition, one could knock down VEGFR2 or VEGFR3 independently with siRNA to determine the differential effects of VEGF-C on LECs through each receptor.

The role of the Angiopoietin-Tie pathway has been well established as a regulator of lymphatic junctions, specifically Angpt2 acting as a ligand on the Tie2 receptor. It is known that Angpt2 is secreted from endothelial cells of both blood and lymphatic vessels and acts in an autocrine fashion, activating the endothelial cells that generates it (Gale et al., 2002; Wang et al., 2022). Angpt2 is often known to act as an agonist or antagonist to Tie2 in a context-dependent manner (Gale et al., 2002; Wang et al., 2022). However, mice lacking Angpt2 have severe lymphatic defects, including poor lymphatic uptake of Evans blue dye, which can be corrected with the addition of Angpt1, a known Tie2 agonist (Gale et al., 2002; Dellinger et al., 2008; Eklund et al., 2017; Wang et al., 2022). This suggests that, in the context of lymphatic vascular activation, Angpt2 acts mainly as a Tie2 agonist (Gale et al., 2002). Angiopoietins are also known to induce both angiogenesis and lymphangiogenesis (Wang et al., 2022), but Gale et al. proposed that the VEGF family is more vital for early stages of vessel growth while Angiopoietins are required for the stages of vascular remodeling and maturation (Gale et al., 2002). Tie2 agonism leads to eNOS activation in a Protein Kinase B (Akt) dependent mechanism, leading to VE-cadherin phosphorylation at tyrosine residue 685 (Y685) which is associated with destabilization and increased vascular permeability (Zheng et al., 2014; Eklund et al., 2017; Wang et al., 2022). Zheng et al. determined that Angpt2 is more specific to lymphatic VE-cadherin regulation, being able to simultaneously maintain the integrity of collecting vessel zippers and ensure the proper remodeling of growing initial lymphatics to convert from zippers to buttons (Zheng et al., 2014). Mice treated with anti-Angpt2 were unable to perform lymphatic drainage of fluorescent tracers, suggesting the inability to regulate zipper-to-button transformation in initial lymphatics can impede lymphatic drainage of fluids and macromolecules (Zheng et al., 2014). Therefore, it seems that Angpt2 is an essential molecule that promotes the differential regulation of lymphatic junctional morphology in both the initial and collecting lymphatic vessels to maintain their specialized functions (Zheng et al., 2014). Of particular interest, the mechanism behind Angpt2 maintenance of zipper integrity on lymphatic collecting vessels has no clear downstream path. Further analysis is needed to fully understand how one molecule can have drastically different effects on different segments of one continuous lymphatic vessel.

**FIGURE 2:**
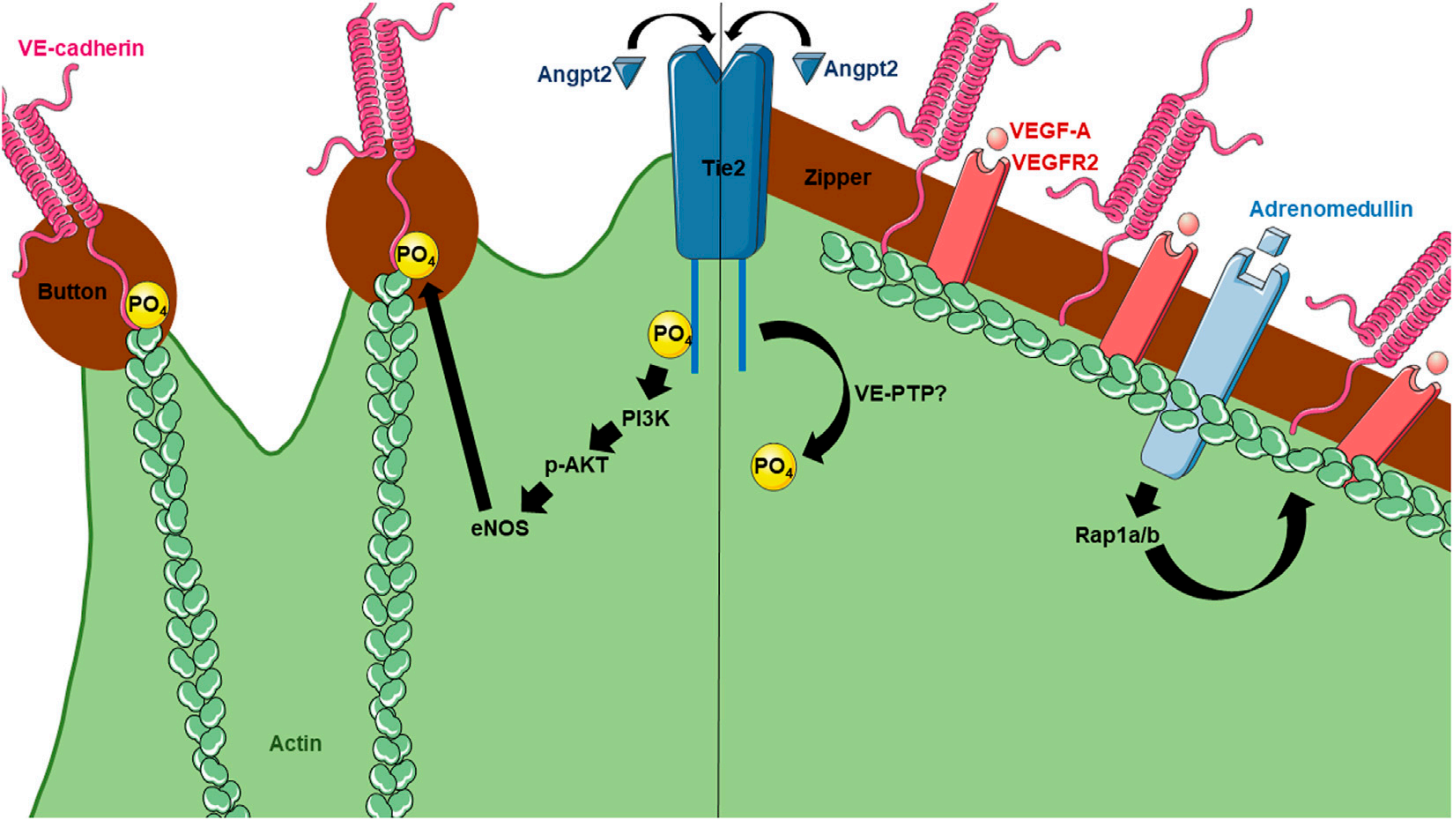
Current knowledge of molecular mechanisms regulating lymphatic button and zipper formation. Angpt2 has been found to affect both button and zipper formation. For button formation (left), Angpt2 activates Tie2 receptors on LECs, leading to its phosphorylation and activation of PI3K, AKT, eNOS, and eventually phosphorylation of VE-cadherin. For zipper formation (right), Angpt2 becomes dephosphorylated by unknown mechanisms. However, VEGF-A has been shown to induce zippering in the skin and intestine, but not the lungs. Adrenomedullin also contributes to zipper formation.

One possible downstream molecule could be Vascular Endothelial Protein Tyrosine Phosphatase (VE-PTP) which has been shown to negatively regulate Tie2 signaling by promoting its dephosphorylation while also associating with VE-cadherin to promote endothelial junction integrity (Eklund et al., 2017). Current knowledge of VE-PTP is limited in the lymphatic endothelium, especially as it relates to functional lymphatic flux (Vockel and Vestweber, 2013; Deng et al., 2015). It is important to note that other junctional proteins could be involved in the regulation of buttons and zippers. In particular, adrenomedullin is a molecule that has been shown to stabilize zonula occludens-1 (ZO-1) and VE-cadherin, decreasing the rate of lymphatic uptake from the interstitium (Dunworth et al., 2008). This is possibly due to induction of continuous and zippered junctions in the initial lymphatic vessels, preventing uptake. Later, Rap1a/b was found to be an important downstream effector of adrenomedullin induced zipper formation during development (Xu et al., 2018). Figure 2 provides a summary of the various molecules that have been shown to alter lymphatic junctional morphology, as described in detail above.

## 6 Lymphatic permeability and flux in cardiovascular diseases

There is substantial evidence that lymphatic dysfunction often contributes to cardiovascular disease pathogenesis, and enhancing lymphatic functionality may improve some of these diseases (Brakenhielm and Alitalo, 2019; Hu et al., 2019; Balasubbramanian and Mitchell, 2022; Harris et al., 2023; Liu et al., 2023). Patients suffering with lymphatic diseases such as lymphedema or lipedema are significantly more likely to develop cardiovascular comorbidities later in life, further linking lymphatic dysfunction with cardiovascular health (Rockson et al., 2022). Despite this, our knowledge of lymphatic junctional morphology in the cardiovascular system is limited for both healthy and diseased states. Herein, we present studies investigating the lymphatic system’s role in atherosclerosis and cardiac pathologies, focusing on the contribution of lymphatic permeability in these diseases. Cardiovascular diseases are often associated with inflammation which has altered lymphatic junctional morphology in other organs. Therefore, the inability to transport macromolecules and immune cells due to zippering of initial lymphatics or buttoning of collecting lymphatics would prevent efficient lymphatic flux that would otherwise contribute to disease resolution (Brakenhielm et al., 2020).

### 6.1 Atherosclerosis

When arteries become damaged from the accumulation of oxidized low-density lipoprotein (oxLDL), monocytes are recruited to the subendothelial space where they become macrophages that engulf the oxLDL, contributing to localized vascular inflammation and the formation of foam cells which leads to atherosclerotic plaque formation (Falk, 2006; Björkegren and Lusis, 2022). This pathophysiology contributes to stroke, myocardial infarction, and abdominal aortic aneurysms (Björkegren and Lusis, 2022; Roldán-Montero et al., 2022). To prevent the downstream effects of this pathology, research has focused on decreasing LDL cholesterol, dampening inflammation, and increasing reverse cholesterol transport (RCT). RCT is the process where cholesterol is recycled into high density lipoprotein (HDL) which mainly travels through the lymphatic vessels and makes its way back to the liver where it can finally be recycled into bile acids or be excreted in the feces (Veronique and Ying, 2013; Randolph and Miller, 2014; Huang et al., 2015; Ouimet et al., 2019; Feng et al., 2022). An early paper by Martel et al. determined that RCT occurs through adventitial lymphatic vessels of large arteries (Martel et al., 2013). Later studies also showed that mouse strains with impaired lymphatic vessel function had increased atherosclerosis when bred into an atherogenic strain (Vuorio et al., 2014).

To date, no studies have determined the junctional morphology of initial or collecting lymphatic vessels in the adventitia of atherosclerotic aortae, leaving our understanding of this topic under-characterized. Regardless, oxLDL and the formation of reactive oxygen species that ensues in endothelial cells is hypothesized to lead to alterations in lymphatic permeability (Zmijewski et al., 2005; Singla et al., 2022). Although not in atherosclerosis, one study indicated that oxLDL induced decreased lymphatic permeability associated with increased VE-cadherin zipper formation (Burchill et al., 2021).

Atherosclerosis development is associated with an increase in adventitial lymphatic vessels, though it is controversial if this lymphangiogenesis is compensatory or disease promoting (Yeo et al., 2020). Subsequent plaque regression, associated with lipid-lowering ezetimibe treatment, leads to reduced lymphangiogenesis comparable to that of mice pre-atherosclerosis (Yeo et al., 2020). Even though increased adventitial lymphangiogenesis is associated with atherosclerosis, there is decreased immune cell egression through lymphatics, contributing to the pro-inflammatory environment of the plaque (Yeo et al., 2020). During chronic inflammation, lymphatic dysfunction is hypothesized to be associated with the formation of tertiary lymphoid organs due to inefficient lymphatic drainage of immune cells (Rehal and von der Weid, 2017; Reed et al., 2019). Therefore, it may be no surprise that atherosclerotic plaques have been associated with the formation of adventitial tertiary lymphoid organs (ALTO) (Mohanta et al., 2014; Yin et al., 2016; Akhavanpoor et al., 2018). Altogether, it is important to note that adventitial lymphatic vessels could play a dual role in reducing atherogenesis by improving reverse cholesterol transport and modulating immune cell egression (Ravaud et al., 2021; Yeo et al., 2021). It is reasonable to assume that this may be mediated, at least in part, by proper control of lymphatic junctional morphology to promote efficient lymphatic flux (Bauer et al., 2022). However, there is contradictory data surrounding the efficiency of immune cell transmigration through the gaps between buttons on initial lymphatic vessels (Zhang et al., 2020).

Athero-prone mouse models also have lymphatic defects, including leakage of Evans blue dye and decreased immune cell migration through lymphatics (Milasan et al., 2016). This suggests possible button junction formation in collecting vessels, preventing their ability to maintain lymph within the system. The authors also saw enhanced lymphatic drainage in collecting vessels of athero-protected *Pcsk9* ^*−/−*^ mice (Milasan et al., 2016). After analysis of initial lymphatic vessels through immunohistochemical analysis of ear and aortic sinus, they concluded no morphological changes, though they did not evaluate the morphology of junctional proteins like VE-cadherin or ZO-1 (Milasan et al., 2016). Other labs have reported lymphatic vessel dysfunction in athero-prone mice of other genetic backgrounds such as *Apoe* ^*−/−*^ (Lim et al., 2009). Further analysis determined multiple deficits in collecting lymphatic vessel function, including hyperpermeability (Davis et al., 2022). Clearly, more research in this area is needed.

### 6.2 Other potential cardiac pathologies affected

Ischemic strokes occur from *in situ* atherosclerotic plaque obstruction in cerebral arteries or migration of an embolism from distant atherosclerotic plaque ruptures to occlude the cerebral arteries (Kim and Caplan, 2016). The draining lymphatics of the brain parenchyma and meninges are the glymphatics and meningeal lymphatics, respectively. The glymphatic system is a lymphatic analog in the periarterial and perivenous space, lacking the classic architecture of a lymphatic vessel (Rasmussen et al., 2018). On the other hand, the meningeal lymphatics form classical vessels that line the venous sinuses (Yanev et al., 2020). Recent studies suggest dysfunction of both the glymphatic and meningeal lymphatic systems in during stroke pathogenesis, albeit lacking analysis of junctional formation relating to lymphatic function (Yanev et al., 2020; Lv et al., 2021; Toh and Siow, 2021). However, initial lymphatic vessels in the meningeal lymphatic system have discontinuous VE-cadherin junctions representing button-like formation (Louveau et al., 2018). Further analysis of meningeal lymphatic junctional morphology and lymphatic flux throughout ischemic stroke progression could yield interesting results.

Myocardial infarction (MI) also occurs as a result of end-stage atherosclerosis which leads to plaque vulnerability and rupture with subsequent thrombosis and occlusion of a coronary vessel (Björkegren and Lusis, 2022). It is still unclear as to whether cardiac lymphangiogenesis is vital immediately after infarction occurs, but it may impact the heart’s recovery in the long term (Oliver et al., 2020; Klaourakis et al., 2021; Liu et al., 2023). An early study found aberrant lymphatic remodeling and decreased lymphatic flux associated with MI, while induction of lymphangiogenesis leads to decreased duration of inflammation (Klotz et al., 2015; Henri et al., 2016; Vieira et al., 2018). Other studies in zebrafish also support the importance of immune cell efflux through lymphatics, which may rely on junctional proteins as well as chemokine expression in lymphatic endothelial cells, to improve function post-MI (Houssari et al., 2020). However, limited studies thoroughly evaluate lymphatic function through permeability in MI or other cardiac pathologies.

Upon lymphatic-specific deletion of VE-cadherin, there is lymphatic vessel regression leading to discontinuous vessels which contributes to collecting vessel permeability and increased heart water content at baseline (Harris et al., 2022). After MI, the lymphatic specific VE-cadherin knockout mice have increased infarct size and fibrosis, but surprisingly they do not have differences in function when compared to control MI mice (Harris et al., 2022). The same lab also showed that adrenomedullin stabilizes the lymphatic endothelial cell barrier, decreasing permeability (Dunworth et al., 2008). Although they did not analyze junctional morphology *in vivo*, the decreased permeability is possibly from promoting continuous zipper-like morphology of junctions in initial lymphatics which would perpetuate inflammatory edema (Dunworth et al., 2008). They later showed that adrenomedullin also targets Connexin43 (Cx43), a gap junction protein, to seemingly promote junctional zipper morphology (Trincot et al., 2019). Despite this, adrenomedullin improved cardiac function and suppressed cardiac edema post-MI (Trincot et al., 2019). Therefore, it is important to study the changes in the junctional morphology of initial and collecting lymphatics of the heart in health and post-MI to gain a better understanding of the regulation of lymphatic flux in this complex disease.

Cardiac inflammation and/or edema is present in many more cardiac pathologies, including but not limited to, ischemia reperfusion injury, diabetic cardiomyopathy, Kawasaki disease, and Takotsubo cardiomyopathy (Brakenhielm and Alitalo, 2019). In addition, natriuretic peptides ANP and BNP are released during various cardiac pathologies and have been shown to increase lymphatic collecting vessel permeability *ex vivo*, which would also lead to decreased lymph flux (Scallan et al., 2013). Therefore, there are endless possibilities to the disease-specific effects on lymphatic junctional regulation and lymph flux.

## 7 Conclusion

When taken together, current research shows a beneficial result from increasing lymphangiogenesis during inflammatory syndromes, including cardiovascular diseases. However, a more vital aspect of lymphangiogenesis is the subsequent function, particularly the permeability, of the newly formed vessels. Permeability is dynamically regulated by junctional morphology during development, health, and disease processes. Maintaining the appropriate permeability in both initial and collecting lymphatic vessels can ensure that lymph enters and remains within the system, contributing to efficient lymphatic flux. Proper permeability and lymphatic flux could benefit cardiovascular diseases by improving white blood cell egression, reverse cholesterol transport, and potentially other lymphatic functions. While current research suggests that the regulation of lymphatic buttons and zippers are vital to lymphatic function, we do not know 1) how a single molecule Angpt2 can orchestrate both button and zipper formation throughout one continuous lymphatic vessel, 2) the molecular mechanisms determining LEC junctional conversion from buttons to zippers during inflammation, and 3) how junctional morphology and subsequent lymphatic function is altered in other inflammatory diseases such as atherosclerosis. Therapeutic modalities which can regulate lymphangiogenesis and/or lymph flux hold promise as novel therapeutic opportunities to treat cardiovascular diseases. Future research should determine the molecular mechanisms that maintain the differential regulation of lymphatic junctions as it relates to lymph flux in health and disease states.
